# Perspective: Biochemical and Physical Constraints Associated With Preparing Thin Specimens for Single-Particle Cryo-EM

**DOI:** 10.3389/fmolb.2022.864829

**Published:** 2022-04-26

**Authors:** Bong-Gyoon Han, Max Armstrong, Daniel A. Fletcher, Robert M. Glaeser

**Affiliations:** ^1^ Lawrence Berkeley National Laboratory, University of California, Berkeley, Berkeley, CA, United States; ^2^ Department of Bioengineering, University of California, Berkeley, Berkeley, CA, United States; ^3^ Biological Systems and Engineering Division, Lawrence Berkeley National Laboratory, University of California, Berkeley, Berkeley, CA, United States; ^4^ Chan Zuckerberg Biohub, San Francisco, CA, United States

**Keywords:** cryo-EM, sample thickness, air-water interface, axisymmetric draining, affininity grids

## Abstract

While many aspects of single-particle electron cryo-microscopy (cryo-EM) of biological macromolecules have reached a sophisticated level of development, this is not yet the case when it comes to preparing thin samples on specimen grids. As a result, there currently is considerable interest in achieving better control of both the sample thickness and the amount of area that is useful, but this is only one aspect in which improvement is needed. This Perspective addresses the further need to prevent the macromolecular particles from making contact with the air-water interface, something that can result in preferential orientation and even structural disruption of macromolecular particles. This unwanted contact can occur either as the result of free diffusion of particles during the interval between application, thinning and vitrification of the remaining buffer, or—when particles have been immobilized—by the film of buffer becoming too thin prior to vitrification. An opportunity now exists to apply theoretical and practical insights from the fields of thin-film physical chemistry and interfacial science, in an effort to bring cryo-EM sample preparation to a level of sophistication that is comparable to that of current data collection and analysis.

## Introduction

Although high-resolution electron cryo-microscopy (cryo-EM) of purified biological macromolecules (Glaeser et al., 2021) has become a successful and mature method, preparing the required thin, vitrified samples often remains a major challenge. Efforts have been under way for some time to improve the way in which samples are thinned before they are vitrified, as has recently been reviewed by Weissenberger et al. (2021). Nevertheless, the classic goal of embedding particles within free-standing films of buffer, as depicted in the cartoon shown as Figure 2 of (Glaeser, 2021), appears to be rarely achieved. Instead, the desired encapsulation of biological macromolecules within vitrified buffer is generally thwarted by rapid diffusion of proteins to the air-water interface (AWI), often followed by formation of a sacrificial, denatured-protein monolayer (Han and Glaeser, 2021). While subsequent adsorption of additional particles to such sacrificial monolayers may still result in an acceptable outcome, it often does not, and better alternatives are generally needed.

Interaction of proteins with the AWI was considered to be a potential hazard in the early days of cryo-EM sample preparation—see section 6.6 of (Dubochet et al., 1988). Indeed, attempts were made at that time to develop some type of electron-transparent slide and coverslip that might be suitable for use in cryo-EM. Exploratory directions of work included sandwiching samples between thin, hydrophilic support films (Taylor and Glaeser, 1973; Taylor and Glaeser, 1976) and the use of fatty-acid monolayers as a conformal coverslip (Hayward et al., 1978; Chang et al., 1985a; Chang et al., 1985b).

Nevertheless, the simplicity and the success of blotting a holey support film with filter paper initially caused such concerns to be put aside.

The issue was reopened, however, in a retrospective of cryo-EM sample preparation—see Figure 5 in (Taylor and Glaeser, 2008). Awareness then continued to grow that the challenges encountered when preparing samples on grids were due to interaction with the AWI, rather than to how the sample was isolated. Perhaps the foremost indication was that the number of particles seen in images was often either more than, or fewer than, what would be expected from the known sample concentration and the thickness of the sample (Vinothkumar and Henderson, 2016). Finally, after cryo-tomography demonstrated that nearly all types of biological macromolecules were adsorbed to the AWI (Noble et al., 2018; D'Imprima et al., 2019; Fan et al., 2019), the problem again began to be discussed more widely in the literature (Drulyte et al., 2018; Carragher et al., 2019; Klebl et al., 2020). It now is widely acknowledged that avoiding interaction with the AWI remains one of the most important challenges still to be solved for single-particle cryo-EM.

This Perspective focuses on the strategy of immobilizing particles onto the surface of a thin support film, preferably followed by washing off unbound particles with buffer, and then removing all but a suitably thin layer of the wash-buffer. Other potential strategies for avoiding interaction with the AWI, which are not reviewed here for lack of space, include 1) thinning and then vitrifying samples so quickly that adsorption to the AWI does not have time to occur; 2) sandwiching samples between structure-friendly, electron-transparent windows, in effect confining particles between some type of electron-transparent “slide and coverslip” (Frederik et al., 1989); and 3) milling or sectioning thin samples from bulk-frozen material.

More specifically, this Perspective addresses two issues that remain relevant after macromolecules have been immobilized onto the surface of a grid. The first of these is the fact that immobilization does nothing that might reduce the unwanted variation in ice thickness that is produced by traditional blotting with filter paper. Second, although immobilization prevents diffusion of particles to the AWI, it does nothing to prevent the AWI from still touching the particles, should the thickness of buffer become comparable to, or less than, the size of the bound particles themselves.

## Immobilization of Particles can be an Effective Way to Avoid Initial Contact With the AWI

Some macromolecules will hit and perhaps adsorb to the AWI, even while the drop of sample is initially forming at the tip of the pipette, because particles that are within 100 nm of a newly formed aqueous surface will diffuse to the AWI within a ms or less (Taylor and Glaeser, 2008; Naydenova and Russo, 2017). Something similar is expected to happen when protein solutions wet the surface of a dip pen, and subsequently the grid surface, a system that is roughly equivalent to the glass rod historically used to quantitatively deliver denatured protein to the surface of a Langmuir trough (Trurnit, 1960). In addition, diffusion will deliver particles to the fresh AWI that then spans the exposed side of ∼micrometer-sized, open holes of the holey carbon grid. In other words, the AWI on the underside (i.e., back) of the grid is just as much of a hazard to proteins as is the larger, continuous surface over the top of the deposited sample.

As mentioned in the Introduction, one alternative is to bind samples onto the surfaces of support films. Furthermore, excess sample might be washed from such grids, depending about the binding affinity, the intent being to remove proteins adsorbed to the AWI at the top of the applied sample. There are, in fact, many ways in which biological macromolecules can be immobilized at solid-liquid interfaces in a structure-friendly way, as is known from the field of biochemical chromatography. Grids that are intended to immobilize biological macromolecules in a structure-friendly way are therefore referred to here as being “affinity grids”, a terminology that is meant to be taken figuratively rather than literally.

At the same time, there are also many types of solid substrates that are not always structure friendly, as has been reviewed briefly in the Discussion section of (Joppe et al., 2020). Thus, as is summarized in Table 1, adsorption onto solid surfaces such as glow-discharge treated carbon film or graphene oxide may produce favorable results for some proteins, while adsorption of other proteins to the same substrates results in preferred orientation or even severe particle damage. It therefore seems likely that the same, specimen-dependent outcome will prove to be true for adsorption of proteins onto the silicon nitride windows of microfluidic EM grids (Huber et al., 2022).

**TABLE 1:** Representative examples of different types of affinity grids used for cryo-EM sample preparation, and the current status of results that have been obtained.

Type of support film	Expected features Risk of damage or preferred orientation	Binding strength	Results Selected references
Glow-discharge treated carbon film	• Although the surface is polar, some types of particles may still be damaged upon binding and other types may still show preferred orientation	• Particle -dependent; varies from weak to strong	• Although this may the first thing to try if sample preparation proves to be challenging, and numerous high-resolution structures have been obtained with such support films, evaporated carbon contributes a level of structural noise that may be undesirable, especially for small particles
Functionalized carbon film	• Provides improved specificity with which particles are bound; risk of preferred orientation remains possible	• Usually intermediate in strength	• Methods of functionalization include nonspecific pre-binding of antibodies (Yu et al., 2016) and designed chemical modification of the surface (Llaguno et al., 2014)
Graphene oxide	• Although the surface is polar, some types of particles may still be damaged upon binding and others may still show preferred orientation	• Usually intermediate in strength	• Although many high-resolution structures have been obtained with graphene oxide, there is a trade-off between covering a high percentage of holes and limiting the number of graphene oxide flakes that lie over individual holes
Functionalized graphene or graphene oxide	• While both ionic binding and chemically specific binding has been achieved, there also still remains a risk of damage or preferred orientation unless the surface is further passivated, for example by additional functionalization with polyethylene glycol	• Binding varies from weak to intermediate	• Physisorption of aromatic groups that bear ionizable groups; high resolution achieved for fatty acid synthase (D'Imprima et al., 2019)
			• Covalent modification with Ni-NTA functional groups; high resolution achieved for streptavidin (Liu et al., 2019)
			• Covalent functionalization of components of the Spy/SpyCatcher affinity tag system; high resolution achieved for a Hsp90 chaperone particle that previously resisted specimen preparation (Wang et al., 2020)
			• Glow discharge deposition of ionizable precursor gasses; high resolution achieved for 30S ribosomal particles and for apoferritin (Naydenova et al., 2019)
Monolayers of charged lipids and ligand-functionalized lipids	• Provides excellent control of the type of charged group and the surface-charge density or, alternatively, the type of ligand to present for binding	• Binding varies from weak to intermediate	• Multiple successes were achieved for growth of monolayer protein crystals (Taylor et al., 2007), but not yet productively used for making single-particle specimens; for more on the latter methodology see (Kelly et al., 2010a; Kelly et al., 2010b) and (Benjamin et al., 2016)
Streptavidin monolayer crystals	• Combines exceptionally tight binding and complete passivation of the interface; little risk of preferred orientation when lysine residues are randomly biotinylated	• Strong	• Four high-resolution structures have been obtained for protein complexes that had been refractory to all previous methods tried when making grids for cryo-EM: RNAP-II elongation complex (Lahiri et al., 2019); polycomb repressive complex in complex with co-factors and histones (Kasinath et al., 2021); phycobilisomes (Sauer et al., 2021), and cytoplasmic dynein-1 (Gillies et al., 2021)

A large number of alternatives have already been investigated for making affinity grids, as is summarized in Table 1. Some of those strategies have already given high-resolution results, even when using samples that previously resisted preparation with standard approaches (D'Imprima et al., 2019; Gillies et al., 2021; Kasinath et al., 2021; Lahiri et al., 2019; Sauer et al., 2021; Wang et al., 2020). While all such affinity grids have attractive features, the most appealing may be ones with high binding affinities, so that—as mentioned above—grids can be washed without eluting the immobilized particles. Furthermore, it is essential that there be little risk that the chosen immobilization strategy results in preferred orientation of particles. The point being made here is that many effective strategies are already available for making affinity grids, and it is certainly welcome if more of them can be developed.

## New Vitrification Strategies Should be Considered When Particles are Immobilized Onto Affinity Grids

As previously indicated, one can blot affinity grids with filter paper in the traditional way, but the usual, unsatisfactory features produced by blotting will still remain. These include the fact that some sample often finds its way to the back side of grids during blotting (Armstrong et al., 2020); there are large variations in the amount of area over which the sample is effectively opaque to 300 keV electrons; there often are many areas in which the ice may seem to be relatively transparent to the electron beam, but it is still not yet thin enough to get the best result; and there are other areas where the ice is either too thin or where the buffer has even dewetted the support film, causing those areas of the grid to dry before vitrification.

Newer alternatives to blotting with filter paper, which are intended to overcome at least some if not all of these shortcomings, have recently been reviewed in (Weissenberger et al., 2021). These alternatives include different ways in which samples are sprayed onto grids, which can be either self-wicking grids (Wei et al., 2018) or conventional holey-film grids, as well as ways in which samples are spread with either a capillary (Arnold et al., 2017) or a dip pen (Ravelli et al., 2020). However, since those methods are unlikely to be compatible with a washing step, it is hard to imagine ways to remove unbound material when using affinity grids. As a result, there still is reason to seek alternative ways to produce thinned films on affinity grids.

Among the alternatives that seem to have not yet been investigated, one might think of applying some type of body force, such as the inertial force employed in spin coating (Larson and Rehg, 1997), or using a strong air flow to “blow off” unwanted buffer. Other possibilities might include creation of a gradient of surface tension from one edge of a grid to the other in order to generate Marangoni flow (Velarde and Zeytourian, 2014), or mechanically squeezing excess buffer from the sample with an electron-transparent “coverslip”, as was attempted in some of the early work described above.

In addition, conspicuous by its near-absence from the cryo-EM literature, is the idea of simply “wicking” excess buffer by touching filter paper to the edge of a grid, as is often done during negative staining. That approach is unsatisfactory when wicking is done from one edge, of course, because it leaves behind a spherical cap of liquid that is several micrometers thick (Glaeser et al., 2016). Axially symmetric draining (wicking), on the other hand, has the potential to produce a uniformly thin film across much or all of the grid. This latter approach was referred to as “blotting from the perimeter” (Armstrong et al., 2020), or in the oxymoronic description used here, “blotting with a hole”.

### The Proposal to “Blot With a Hole” has Many Precedents

**FIGURE 1:**
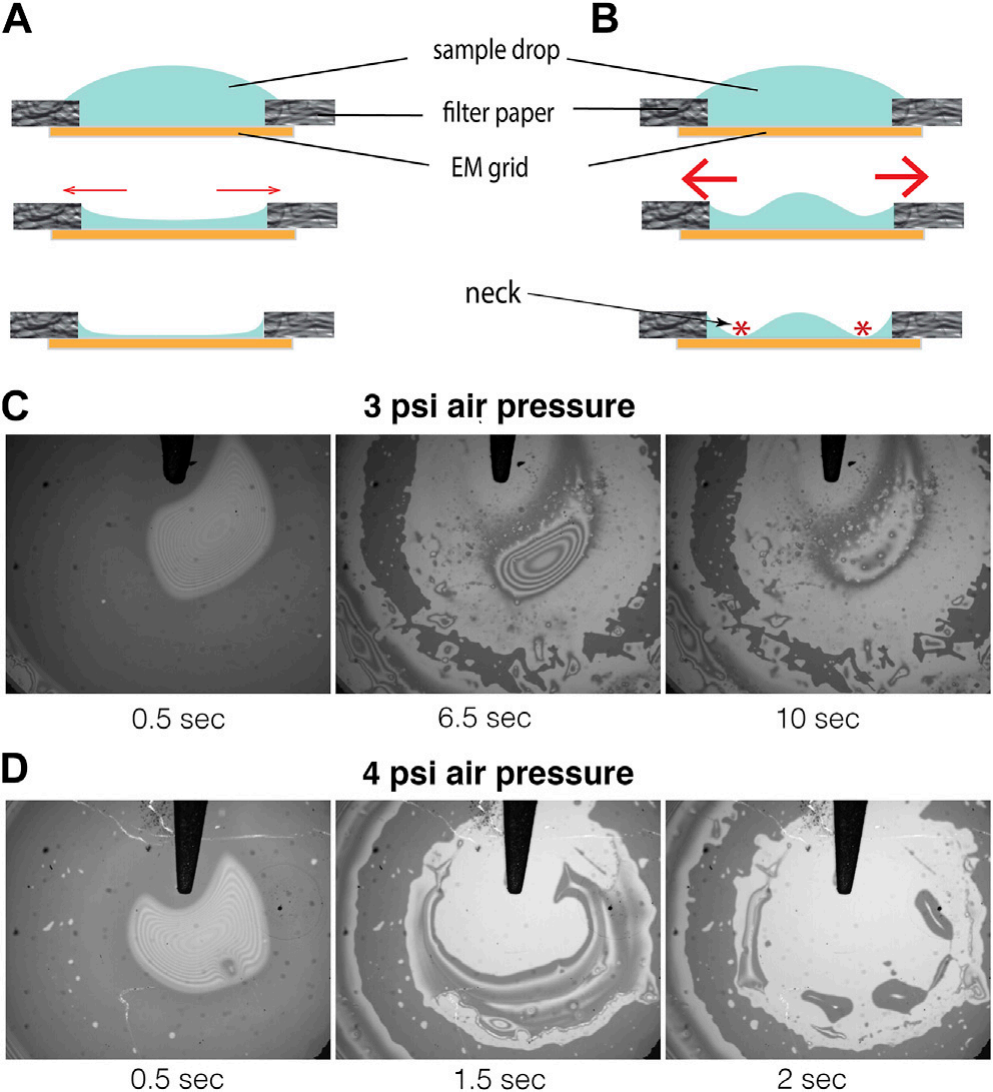
Concept of, and preliminary results obtained by, axisymmetric draining of excess buffer from 3 mm coverslips. **Panel A** shows the desired, ideal outcome if excess buffer is removed adiabatically, indicated by thin red arrows. The sample is expected to become progressively thinner in the center, remaining thick only in the so-called Plateau border, closest to the edge of the filter paper. **Panel B** shows the expected, undesirable result that can occur if buffer is removed too quickly, indicated symbolically by thicker red arrows, thereby producing a thin neck that isolates a too-thick puddle that remains in the middle of the hole. **Panels C** and **D** show two example of preliminary reflection interference contrast microscopy (RICM) results obtained when a gentle stream of humid air was used as an additional driving force to mitigate the problem of necking. A mottled, dark ring remains after most of the buffer has been removed, which corresponds to the still-lubricated regions between the coverslip and the filter paper. Different values of the air pressure were empirically adjusted at the source to values of 3 psi in panel C and to 4 psi in panel D, respectively. The thickness of the buffer, if any, that remains in the extensive, relatively bright areas must be significantly less than 100 nm, as is inferred from the absence of interference fringes.

For clarity, the concept of draining excess buffer from the perimeter of an affinity grid is illustrated here by the cartoon shown in Figure 1A **.** The idea to “wick” (drain) sample in an axisymmetric manner is similar to the one used to form a free-standing, thin-liquid film in a Sheludko cell. For reference, cartoons describing Shelduko cells can be found in Figure 3 of (Sheludko, 1967), or perhaps even better in Figure 8 of (Mysels, 1964). The Sheludko cell, or one of its many descendants, has long been used to study the thickness-dependent interfacial forces that become relevant when the thickness values of liquid films become less than about 100 nm.

Blotting with a hole in the filter paper can also be described as an attempt to scale up the diameter of the blotted area relative to that achieved when using self-wicking EM grids (Wei et al., 2018). In the latter approach, droplets of sample material are sprayed onto the centers of many grid squares. Next, the droplets spontaneously spread until the sample touches the grid bars, upon which excess liquid is wicked away. Thus, in effect, each grid square of a self-wicking grid can be regarded as being a microscale realization of a Sheludko cell. In practice, of course, the droplets are expected to land at random positions, including some that fall directly on top of grid bars. Fortunately, even then the liquid seems to spread over the open areas of the immediately adjacent grid squares.

Understanding what may be required to produce extended, uniformly thin films by axisymmetric wicking from the perimeter of EM grids involves a number of topics that may not be familiar to most in the cryo-EM community, however. As a result, some relevant background is developed in the following section.

## Principles That Govern the Formation and Stability of THIN, Liquid Films

The principles involved in making samples that are suitable for cryo-EM are the same as those involved 1) in making stable foams (juxtaposed air bubbles), emulsions, and colloidal suspensions, as well as 2) in some technological applications that employ modern microfluidics. As a result, an extensive literature exists that addresses the formation and stability of thin liquid films. Recent reviews (Andrieux et al., 2021; Chatzigiannakis et al., 2021), for example, contain much that can inform our approach to making cryo-EM samples on affinity grids, and an older, more extensive review (Stubenrauch and Klitzing, 2003) is also worth pointing to.

### Thin Liquid Films can Become Unstable Below a Critical Thickness

When a uniform sheet of liquid becomes thin enough, van der Waals interactions begin to exert a pressure that causes the sheet to become even thinner. It may be a surprise to learn that the pressure is inversely proportional to the 3^rd^ power of the film thickness, see equation 7 in (Chatzigiannakis et al., 2021), even though the van der Waals interaction energy between any two atoms is inversely proportional to the 6^th^ power of the distance. The surprisingly long-range nature of the pressure is a result of the fact that the total van der Waals energy is the sum (integral) of all such pair-wise contributions, and there are many more such interactions if a film is thick than if it is thin (Ruckenstein and Jain, 1974). As a result, it becomes energetically favorable for water molecules to move to places where the liquid is thicker. This forces the film to become thinner, i.e., it drives all thin films of liquid in the direction of rupturing. As a result, it is common experience that free-standing bubbles burst, and foams collapse as adjacent bubbles fuse with one another. Similarly, film-rupture and dewetting occurs if a liquid film, supported on a solid, is spread too thinly.

Liquid films can be made to resist rupture, however, if their apposed interfaces exert a repulsive force between one another. If, for example, the air-water interfaces of a free-standing film of buffer are coated by a charged surfactant, electrostatic repulsion increases exponentially as the interfaces approach one another. Even the polar groups of neutral surfactants exert a strong “hydration force” (Parsegian and Zemb, 2011), or osmotic pressure, that resists further thinning. When the repulsive pressure equals the van der Waals pressure to become thinner, a local minimum occurs in the disjoining pressure—see Figure 1B in (Andrieux et al., 2021), which results in a stable film. Repulsive contributions to the disjoining pressure are normally very short-ranged, however, and liquid films may not begin to resist further thinning until their thicknesses fall below a few nanometers—for an example see (Bergeron and Radke, 1992; Yaros et al., 2003).

Soap films are normally stabilized at significantly greater thickness values, however, corresponding to ones that produce interference colors in bubbles. In this range of thicknesses it may be that Marangoni forces, i.e., forces that occur when surface waves generate gradients in surface tension, stabilize films whose thickness values are hundreds of nanometers (Bhamla et al., 2017). Such films are generally too thick to be used for single-particle cryo-EM.

## Preliminary Experiments Have Been Done to Test the Feasibility of Blotting With a Hole

Equipment used previously to observe the removal of excess buffer from 3 mm diameter coverslips (Armstrong et al., 2020) has since been modified, as is described in Supplementary Figure S1, to obtain high-speed movies that show what happens when the same coverslips are blotted with an ∼2 mm hole in filter paper. Blotting pads used for the new experiments were fabricated with a laser-beam etching tool, and these are also described in the Supplementary Figure S1.

### Unwanted Necking can Easily Impede Complete, Axisymmetric Draining

Uniform, axisymmetric removal of buffer, illustrated by the cartoon shown in Figure 1A, is not as easy to achieve as might first be imagined. While the uniformly thin, sought-after profile might be achieved when liquid is removed slowly and reversibly, too rapid a removal is likely to cause the liquid to “neck down” somewhere close to the perimeter, as is illustrated schematically in Figure 1B. Once such a neck becomes very thin, it impedes further removal of liquid from the center. Although many different materials and designs were tried in a first round of experiments, unfavorable results, like those shown in Supplementary Figures S2–S4 proved to be challenging to overcome,.

### Axisymmetric Draining can Be Assisted by Addition of a Humid Air Stream

A second generation of experiments was then undertaken, in which blotting with an ∼2 mm diameter hole was assisted by applying a driving force to the buffer. This was done by directing a stream of humid air through the hole in the filter paper, i.e., onto the sample, as is shown schematically in Supplementary Figure S1B. In addition, a shallow trench, shown in Supplementary Figure S1A, was milled just outside the empty hole to prevent contact between the filter paper and the edge of the coverslip.

After making these changes, the results of blotting with a hole became quite promising. As is shown in Figure 1C, buffer can be removed from nearly all of the area that corresponds to an ∼2 mm diameter hole in the filter paper. A thin film of buffer still remains, which is clearly less than 100 nm in thickness, the value at which a first (dark) interference fringe would appear.

## Discussion

Both the relative humidity and the flow rate are important parameters to control in an air stream that is used to assist axisymmetric draining. Too little flow will still leave a too-large “cap” of liquid at the center; too large a flow might cause the liquid film to rupture and to dewet the substrate; too low humidity will cause excessive evaporation to occur; and too high humidity may produce condensation to form.

Demonstration of the potential usefulness of blotting with a hole thus requires that similar results first be achieved when using EM grids rather than a surrogate, 3 mm coverslip. In addition, such grids would have to be vitrified and examined in an electron microscope, preferably one equipped with an energy filter, so that their thickness values can be measured, for example, by the method described in (Rice et al., 2018). The final proof of usefulness will then depend upon whether the resolution achieved in single-particle cryo-EM maps proves to be as good as, or even better than, that achieved with other grid-preparation methods, assuming that all else remains constant.

While axisymmetric draining seems to be an appropriate approach to use with affinity grids, it may be that it will also be an effective alternative to consider when using standard, holey grids any type of affinity grid could be used, of course, to avoid diffusion to the AWI. Of the options already listed in Table 1, streptavidin affinity grids have the advantage that the resolution retained in the specimen can be easily and unequivocally determined from the highest spatial frequency at which Bragg peaks remain visible in the Fourier transforms of images (Han et al., 2017).

## Data Availability

The datasets presented in this article are not readily available because they refer to work that previously was published to authors of work that is reviewed here. Restrictions do not apply to the datasets. Requests to access the datasets should be directed to RG, rmglaeser@lbl.gov.
