# Supplementary material for: Perspective: Biochemical and Physical Constraints Associated With Preparing Thin Specimens for Single-Particle Cryo-EM
Source: Front Mol Biosci. 2022 Apr 26;9:864829. doi: 10.3389/fmolb.2022.864829 (PMC9100935; doi:10.3389/fmolb.2022.864829)
Supplement: Supplementary file 1 [file DataSheet1.docx]

Supplemental Material

**PERSPECTIVE: BIOCHEMICAL AND PHYSICAL**

**CONSTRAINTS ASSOCIATED WITH PREPARING**

**THIN SPECIMENS FOR SINGLE-PARTICLE CRYO-EM**

Bong-Gyoon Han, Lawrence Berkeley National Laboratory, University of California, Berkeley, CA 94720

Max Armstrong, Lawrence Berkeley National Laboratory, University of California, Berkeley, CA 94720; Department of Bioengineering, University of California, Berkeley, CA 94720

Daniel A. Fletcher, Department of Bioengineering, University of California, Berkeley, CA 94720, Biological Systems and Engineering Division, Lawrence Berkeley National Laboratory, University of California, Berkeley, CA, Chan Zuckerberg Biohub, San Francisco, CA

Robert M Glaeser*, Lawrence Berkeley National Laboratory, University of California, Berkeley, CA 94720

**DETAILS OF THE EQUIPMENT USED FOR PRELIMINARY EXPERIMENTS ON AXISYMMETRIC DRAINING**

All experiments were carried out with a modified Vitrobot, a gift of FEI, now Thermo Fisher Scientific (Eindhoven, NL). The door of the Vitrobot chamber was replaced by a home-built door, which has an access port that allows closer approach by the objective lens of the home-built Reflection Interference Contrast Microscope (RICM). Glass coverslips were held with a standard Vitrobot tweezers, but the latter was attached to the plunger rod with a fixture that allows the coverslip to be viewed face on rather than edge on. The standard blotting paddles were removed and the existing hardware was removed from the ports on both sides of the chamber.


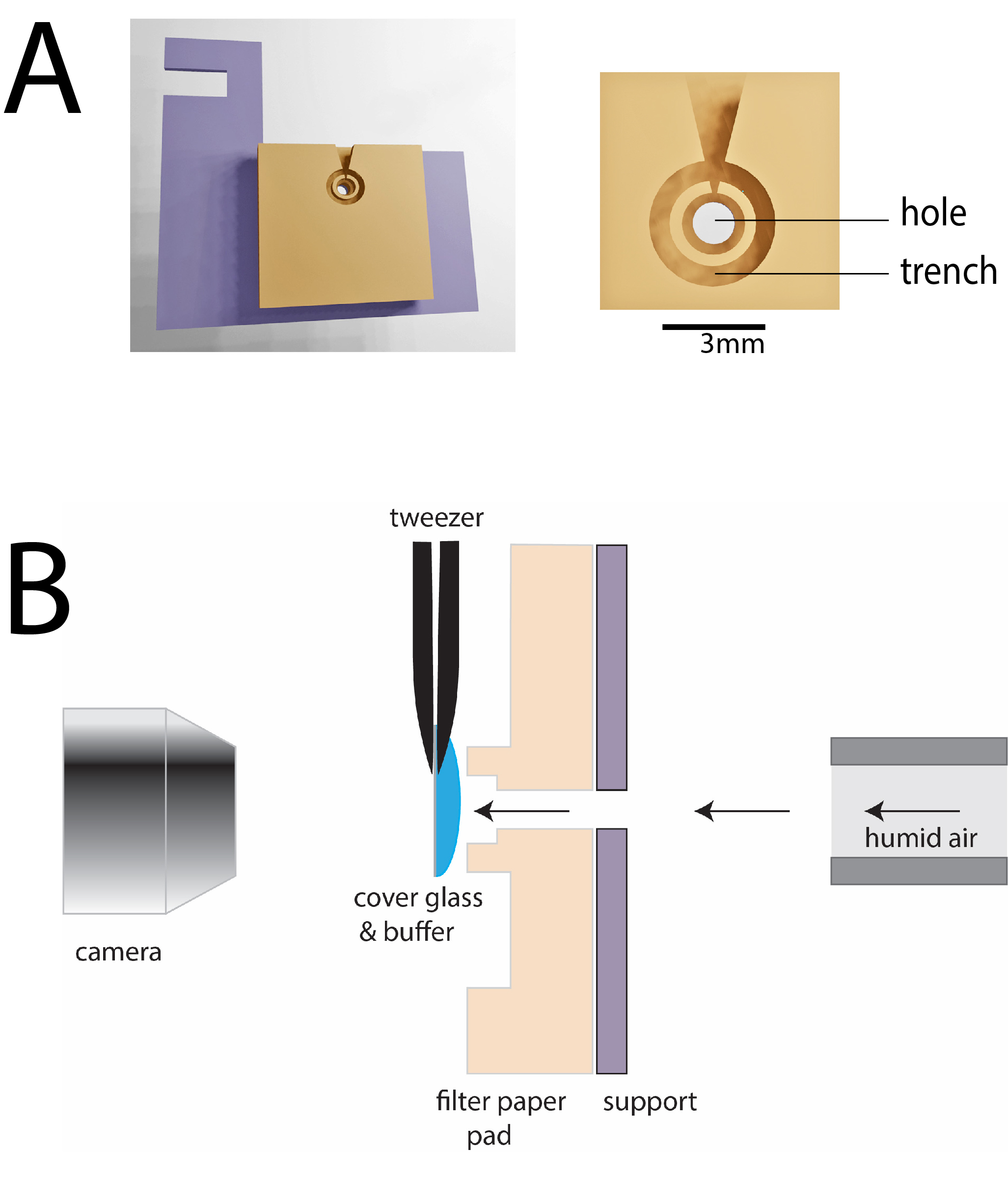


**Figure S1**. Schematic drawing of the equipment used to achieve axisymmetric draining of excess buffer from a 3 mm coverslip, and to record high-speed movies as draining progresses. **A.** The filter paper (tan color), with an ~1.5 – 2 mm diameter hole and also with a circular trench centered at a diameter of ~2.6 mm, was mounted onto a plastic support (purple color). The hook-like detail at the top-left indicates how, in our particular implementation, the support is mounted to a motorized stage. A stack of 15 pieces of filter paper, each 1.5 cm square, was first bonded together with repositionable spray adhesive (3M), and this stack was bonded in the same way onto the rigid support. A laser machining tool, described below, was used to make a hole completely though the filter paper as well as to make the shallow trench, corresponding in thickness to just a few sheets of filter paper. A V-shaped trench was also cut at the top of the filter paper, to accommodate the tip of the tweezer during blotting. **B**. High-speed movies were recorded with home-built RICM optics, as described in (Armstrong et al., 2020), which is represented symbolically in this panel by the objective lens shown on the left. A humid air steam was directed through the hole in the filter paper pad (as well as the rigid support) during blotting, and the circular trench ensured that fibers of the filter paper do not touch the coverslip at its rim.

**High-speed RICM imaging was used to observe the axisymmetric draining process**

The RICM optics and high-speed camera used to observe the draining process remained the same as has been described in the Methods section of (Armstrong et al., 2020). Briefly, the coverslip was illuminated by light that was passed through a 560 nm narrow bandpass filter, and images were captured with a Photron 1024 PCI camera (Photron, Tokyo, Japan).

**Fabrication of the blotting pad**

A stack of 15 pieces of Whatman #1 filter paper, each of them 1.5 cm square, was bonded together to make a pad, as described in the legend for **Figure S1**. This pad was then temporarily bonded to a rigid, custom fabricated support, which was designed to be mounted onto the motorized stage that is described in the following section. Details can be further appreciated by referring to **Figure S1** and its accompanying legend.

A hole of desired diameter, as well as the desired trenches, were made in the filter paper pad with a “Glowforge Basic” tool (Glowforge, Inc., Seattle, WA). Also shown in **Figure S1** is the fact that a hole was also made in the rigid plastic support, in a separate step, to allow the air stream to blow directly onto the buffer that remains on the coverslip.

**A motorized stage was used to advance the blotting pad onto the stationary coverslip as high-speed movies were recorded**

A motor-driven stage, fabricated in the Fletcher lab and installed within the Vitrobot chamber, was used to press the blotting pad against the “front” of a coverslip, i.e. the side of the coverslip onto which a droplet the sample had been deposited. Sample was placed on the coverslip before lifting the tweezer and coverlip into the chamber.

RICM images were recorded from the back side of the coverslip, as indicated in **Figure S1B**. Once the sample is thin enough, these images exhibit fringes produced (1) by light reflected from the interface between the coverslip and the remaining buffer and (2) by light reflected from the air-water interface of the remaining buffer.

**Control over the velocity (pressure) and humidity of the flowing air**

Compressed air was passed through a bottle of water, which was heated to a few degrees Celsius above the temperature of the Vitrobot chamber, in order to ensure that the humidity remained high when air was delivered to the hole in the blotting pad. A gas-flow meter was used to adjust the pressure at the source. This proved to be a satisfactory way to produce reproducible results, even though the pressure was not measured at the coverslip directly.

**PRELIMINARY EXPERIMENTS HAVE BEEN DONE TO TEST THE FEASIBILITY OF BLOTTING WITH A HOLE**

To get a better idea of what the difficulties might be, we undertook preliminary experiments in which a 3 mm glass coverslip was blotted with holes of various diameters and designs.

**Necking causes a puddle of buffer to become isolated from the edge of the filter paper used to drain excess buffer**

While avoiding unwanted necking (described in the main text) may be routinely possible in a properly designed Schelduko-type cell, it proved to be challenging to do so with the limited choice of suction pressure offered by commercial filter papers. Not surprisingly, our initial conclusion was that the limited range of available capillary pore sizes and thicknesses of readily available filter papers provides insufficient control over the sorption pressure and speed that drives wicking. Although a liquid film of uniform thickness could be obtained over a small fraction of the area within the hole, as is shown in **Figure S2**, it was not possible to avoid there being a thick puddle of buffer in the center of a hole, separated from the perimeter by a thin, liquid-film neck.

**
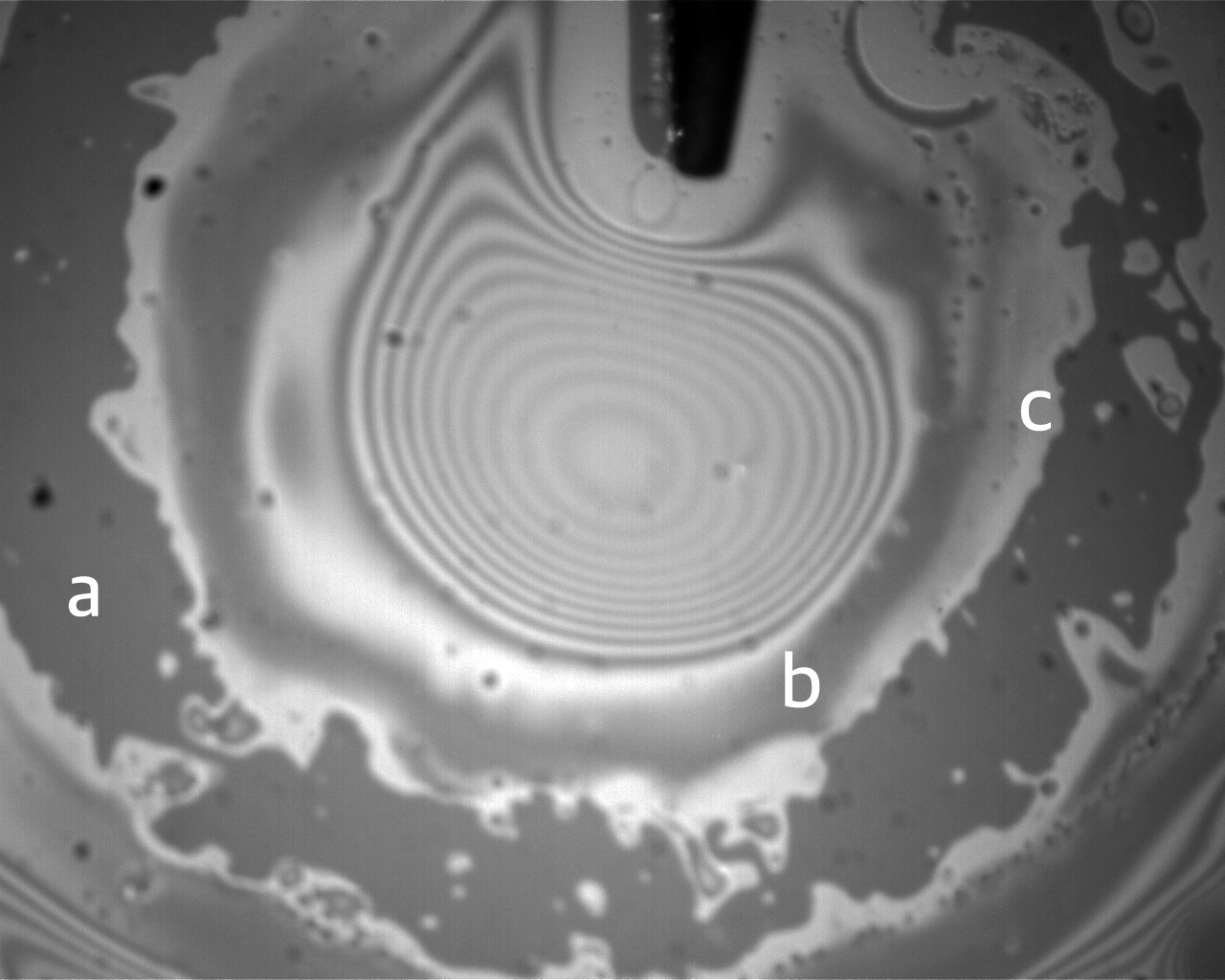
**

**Figure S2**. Example RICM image of the type of liquid films produced (after draining had effectively stopped), obtained without using the pressure from an air stream to apply a driving force to the buffer within the center of the hole in a filter paper. The large, remaining puddle exhibits multiple interference fringes, indicating that it is several hundreds of nm thick over most of its area. Labeled features correspond to **(a)** the well-lubricated contact between filter paper and the front surface of the coverslip; **(b)** first dark interference fringe, corresponding to a water thickness of about 100 nm; **(c)** region in which the water thickness is significantly less than 100 nm, but the film has not dewetted the substrate.

**Buffer leaks onto the back of the coverslip by way of bridges that form**

- **around the tip of the tweezer and**
- **at points were fibers of the filter paper touch the rim of the coverslip**

The above results, although not yet what is needed, were nevertheless instructive because they also revealed some of the ways in which buffer is able to make its way to the back side of a grid during blotting. One long-suspected path to the back of a grid (or in this case a coverslip) was confirmed to be points of contact between filter paper and the rim of the coverslip. **Figure S3** shows an example of buffer having spread to the back via contact between the filter paper and the rim of the coverslip.


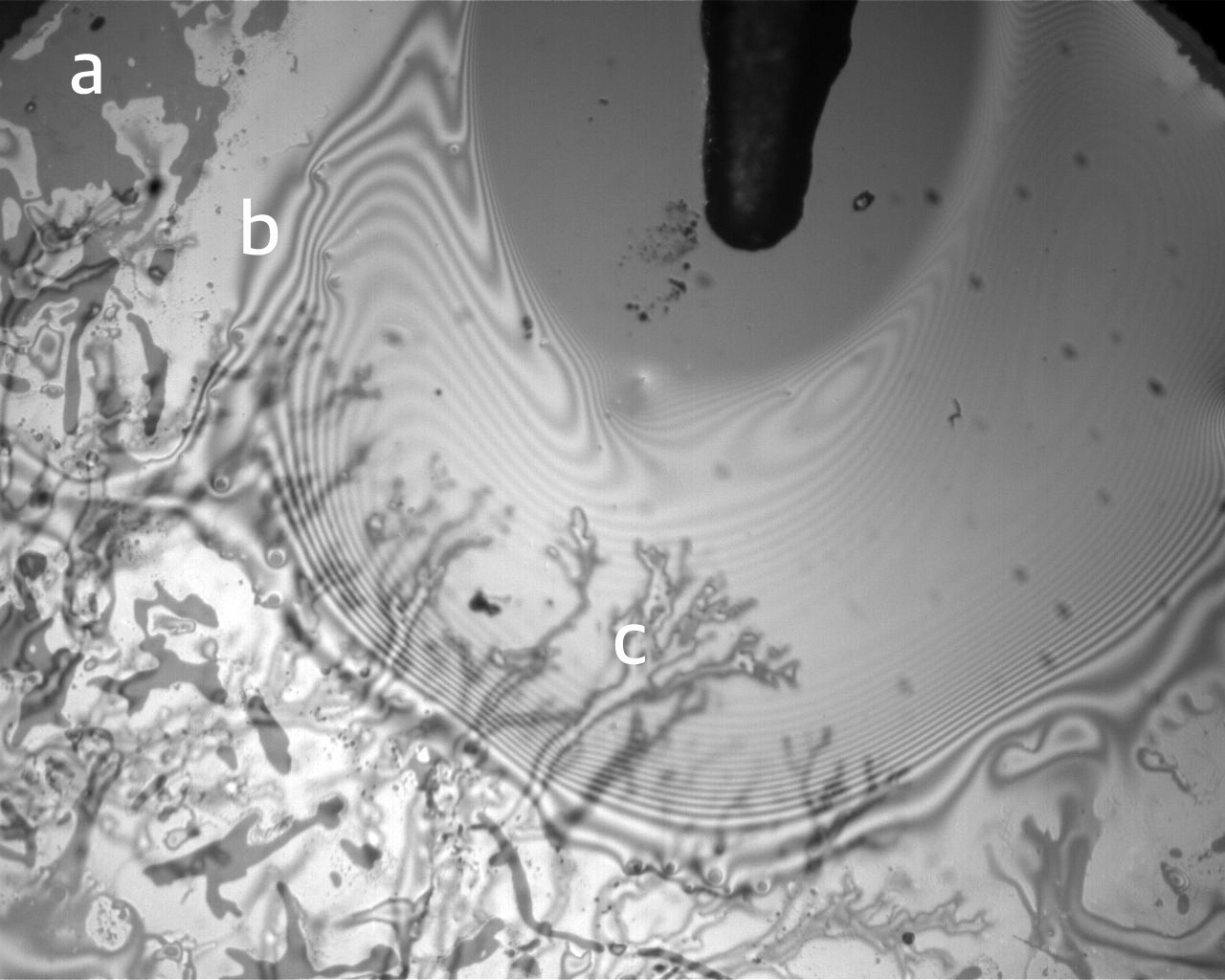


**Figure S3.** Example RICM image of the type of liquid film produced (after draining was effectively complete) when contact between the filter paper and the rim of the coverslip creates a liquid bridge between the front side and the back side of the coverslip. The fact that two, independent sets of interference fringes are seen to be overlapping one another can only be interpreted as being due to buffer being on both sides of the coverslip. Labeled features correspond to **(a)** lubricated contact between filter paper and the front surface of the coverslip; **(b)** first dark interference fringe, corresponding to a water thickness of about 100 nm; **(c)** dendritic fingers of water on the back of the coverslip extend onto the surface of the coverslip.

The wetted tweezer tips can also form a connecting bridge unless care is taken to make them be hydrophobic. **Figure S4** is an example that demonstrates leakage of this type. The residue of such a bridge was previously seen in cryo-SEM images of both sides of an EM grid, shown in (Armstrong et al., 2020).


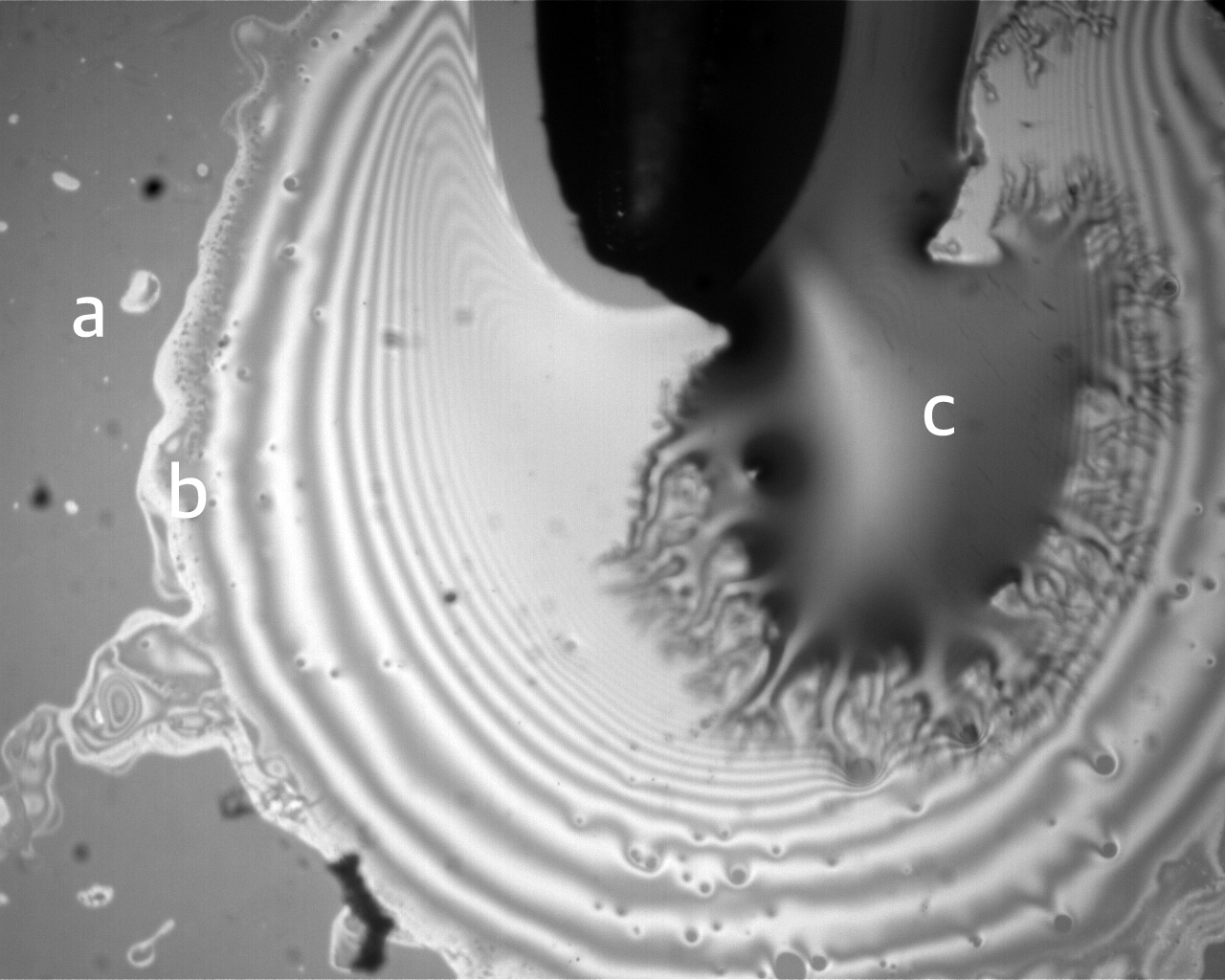


**Figure S4.** Example RICM image of the type of liquid film produced (after draining was effectively complete) when the tip of the tweezers creates a liquid bridge between the front side and the back side of the coverslip. The fact that two, independent sets of interference fringes are seen to be overlapping one another can only be interpreted as being due to buffer being on both sides of the coverslip. Labeled features correspond to **(a)** lubricated contact between filter paper and the front surface of the coverslip; **(b)** first dark interference fringe, corresponding to a water thickness of about 100 nm; **(c)** a puddle of water on the back of the coverslip, from which dendritic fingers extend onto the surface of the coverslip at the advancing line of contact.

Additional pathways might also be possible, through which water can get from the front to the back when using EM grids rather than a coverslip. These include the open holes themselves, in holey-support films, or breaks and tears in a continuous support film.

**REFERENCES**

Armstrong, M., B.G. Han, S. Gomez, J. Turner, D.A. Fletcher, R.M. Glaeser, 2020. Microscale Fluid Behavior during Cryo-EM Sample Blotting. Biophysical Journal 118, 708-719.
